# Ulcer of the Foot Healed by Specifics and Thiersch’s Method of Skin Grafting

**Published:** 1893-08

**Authors:** E. B. Jackson

**Affiliations:** Houston


For Texas Medical Journal.

### ULCER OF THE FOOT HEALED BY SPECIFICS AND THIERSCH'S METHOD OF SKIN GRAFTING.

BY E. B. JACKSON, M. D., HOUSTON,

ON the 24th of December, 1892, after I had made many attempts to heal an ulcer on the foot of a little child two years old, an inmate of the Faith Home, I operated according to Thiersch's method of skin grafting. The ulcer, after its callous edges were cut away, was fully as large as a silver dollar piece, and to fill it up required three slips of skin which I shaved by means of a razor, from the front aspect of the thigh on the healthy limb and maintained them at the body heat, 98½° F., in a salt solution of one drachm to the quart of water. After the hard margins of the ulcer were cut away and its unhealthy bed thoroughly curetted, it was covered by a silk protective and sufficient pressure was made to check the bleeding and dry the raw surface. Now the tissue-paper slips of skin, by means of two probes, were placed into their position with their raw surface facing the raw and purified surface of the ulcer. The foot was now covered with aseptic gauze and put up in a plaster-paris bandage. The dressing was removed five days later, and the skin in the center of the ulcer had taken satisfactory life, but the margins of the ulcer were yet uncovered, which was accounted for by the scantiness of the skin which the little boy's emaciated thigh would only furnish. With careful dressing of the remaining raw edges of the ulcer and with attention to internal treatment, healing was completed in six weeks.

